# 非小细胞肺癌围术期免疫治疗的模式演变：从辅助困境到新辅助优先

**DOI:** 10.3779/j.issn.1009-3419.2026.102.25

**Published:** 2026-08-20

**Authors:** Jie XU, Zhansheng JIANG

**Affiliations:** 1 300060 天津，天津医科大学肿瘤医院，国家肿瘤临床医学研究中心，天津市“肿瘤防治”重点实验室，天津市恶性肿瘤临床医学研究中心，天津市肿瘤免疫与生物治疗重点实验室；肺部肿瘤二科; 1 Department of Thoracic Oncology II; 2 中西医结合科; 2 Department of Integrated Traditional Chinese and Western Medicine, Tianjin Medical University Cancer Institute and Hospital, National Clinical Research Center for Cancer, Tianjin Key Laboratory of Cancer Prevention and Therapy, Tianjin Clinical Research Center for Malignant Tumors, Tianjin Key Laboratory of Cancer Immunology and Biotherapy, Tianjin 300060, China

**Keywords:** 肺肿瘤, 免疫治疗, 新辅助治疗, 辅助治疗, 治疗模式, Lung neoplasms, Immunotherapy, Neoadjuvant therapy, Adjuvant therapy, Treatment pattern

## Abstract

免疫检查点抑制剂（immune checkpoint inhibitors, ICIs）的应用重塑了可切除非小细胞肺癌（non-small cell lung cancer, NSCLC）的围术期治疗格局，目前已形成辅助、纯新辅助及“夹心饼”（新辅助+手术+辅助）三种治疗模式。尽管IMpower010和KEYNOTE-091在辅助免疫治疗中取得阳性结果，但BR.31和ANVIL两项大型III期研究的阴性结局，引发了对该模式的质疑。相比之下，新辅助免疫治疗在多项III期研究中均获得阳性结果，其机制可能在于保留了完整的肿瘤抗原库和引流淋巴结结构，使化疗联合ICIs能够诱导更强的免疫应答。目前纯新辅助和“夹心饼”模式均被指南推荐，但CheckMate-816与KEYNOTE-671的5年总生存（overall survival, OS）率几乎一致（65.4% *vs* 64.6%），且CheckMate-77T的病理完全缓解（pathological complete response, pCR）率（25.3%）与CheckMate-816（24%）亦相差无几，提示“夹心饼”模式中增加新辅助疗程和继续辅助免疫所带来的额外获益或许非常有限。未来，基于pCR和分子残留病灶（molecular residual disease, MRD）的个体化决策模型，如pCR且MRD阴性者降阶梯、non-pCR或MRD阳性者升阶梯，有望优化治疗强度与生存获益的平衡。综上，现有证据倾向可切除NSCLC采用“新辅助优先”策略：在疗效与“夹心饼”模式相当（上述比较主要基于跨研究间接比较）的前提下，纯新辅助模式更具成本效益优势，可作为首选；新辅助后的个体化治疗是未来的发展方向。

## Abstract (en)

The application of immune checkpoint inhibitors (ICIs) has reshaped the perioperative treatment pattern of resectable non-small cell lung cancer (NSCLC). Currently, three treatment paradigms have emerged: adjuvant, neoadjuvant alone, and ‘sandwich’ (neoadjuvant+surgery+adjuvant). Although IMpower010 and KEYNOTE-091 achieved positive results in adjuvant immunotherapy, the negative results of two large phase III studies, BR.31 and ANVIL, raised questions about the generalizability of this model. In contrast, neoadjuvant immunotherapy has shown efficacy in multiple phase III studies. This advantage may stem from the preservation of the intact tumor antigen repertoire and draining lymph node architecture, enabling chemo-immunotherapy to elicit more robust systemic immune responses. Currently, both neoadjuvant alone and ‘sandwich’ models are recommended by guidelines, but the 5-year overall survival (OS) rates of CheckMate-816 and KEYNOTE-671 are nearly identical (65.4% *vs* 64.6%), and the pathological complete response (pCR) rate of CheckMate-77T (25.3%) is comparable to that of CheckMate-816 (24%), suggesting that the incremental benefit of extending neoadjuvant cycles and adding postoperative adjuvant immunotherapy in the ‘sandwich’ model is very limited. In the future, an individualized decision-making model based on pCR and molecular residual disease (MRD) status, such as de-escalation to observation for those achieving pCR with negative MRD, and intensification for those with residual disease or positive MRD, is expected to optimize the balance between treatment intensity and survival benefit. In summary, the existing evidence favors the adoption of a ‘neoadjuvant first’ strategy for resectable NSCLC. Based mainly on cross-trial indirect comparisons, the neoadjuvant alone model offers comparable efficacy to the ‘sandwich’ approach with potential cost-effectiveness advantages, and may be preferred as the initial strategy. Individualized post-neoadjuvant management represents the future direction of this field.

肺癌是全球范围内发病率和死亡率最高的恶性肿瘤，其中非小细胞肺癌（non-small cell lung cancer, NSCLC）约占85%^[1]^。2024年全球癌症统计报告显示肺癌新发病例约248万，死亡病例约181万，仍位居恶性肿瘤首位^[2]^。尽管25%-30%的NSCLC患者在初诊时处于可切除阶段，术后5年无复发生存（recurrence-free survival, RFS）率仍不尽如人意，I期约为82%，III期仅为34%^[3]^。更为严峻的是，根治术后复发多发生在1年以内，且复发类型以远处转移为主，这意味着单纯手术和化疗难以清除术前已存在的微转移灶^[4]^。

免疫治疗的出现为这一困境带来了转机。免疫检查点抑制剂（immune checkpoint inhibitors, ICIs）通过解除T细胞免疫抑制、恢复抗肿瘤免疫应答，已在晚期NSCLC中确立了标准治疗地位。近年来，ICIs逐步向早期可切除NSCLC的围术期领域应用，产生了辅助、纯新辅助及“夹心饼”（新辅助+手术+辅助）三种治疗范式。辅助免疫仅IMpower010^[5,6]^与KEYNOTE-091^[7]^研究取得有限获益，而BR.31^[8]^、ANVIL^[9]^完全阴性；与之相反，所有新辅助相关III期试验均获得阳性终点。这一鲜明对比也使得临床实践在逐渐迈向“新辅助优先”时代。本文将围绕“为何新辅助优先于辅助”这一核心命题，系统梳理相关证据，从临床证据、生物学机制、个体化决策和卫生经济学四个维度系统比较不同模式的优势与局限，为临床决策提供整合性参考。

## 1 辅助免疫治疗：从曙光初现到争议中的反思

### 1.1 早期阳性证据与临床期待

辅助免疫治疗曾被视为解决术后复发问题的理想方案，其逻辑在于：手术已切除原发灶，术后通过ICIs清除循环中可能残留的微小转移灶，从而实现“根治性巩固”。程序性死亡配体1（programmed death ligand 1, PD-L1）的表达水平是辅助免疫治疗人群分层的关键参考指标。IMpower010研究（阿替利珠单抗）^[5,6]^和KEYNOTE-091研究（帕博利珠单抗）^[7]^分别在PD-L1阳性（肿瘤细胞≥1%）和总体人群中观察到无病生存（disease-free survival, DFS）的统计学改善，为辅助免疫治疗进入临床实践提供了关键证据。基于此，美国食品药品监督管理局（Food and Drug Administration, FDA）先后批准阿替利珠单抗和帕博利珠单抗用于可切除NSCLC的术后辅助治疗，标志着辅助免疫治疗时代的开启。然而，这两项研究的获益特征存在显著差异：IMpower010^[5,6]^的获益主要由PD-L1≥50%人群驱动[风险比（hazard ratio, HR）≈0.5]，而PD-L1 1%-49%人群获益有限，PD-L1<1%人群未显示获益；KEYNOTE-091^[7]^虽在全人群中达到统计学差异，但矛盾的是，PD-L1≥50%人群反而未获益，而PD-L1 1%-49%人群观察到获益。PD-L1表达在不同辅助试验中的预测效能存在显著异质性，使其难以作为稳定分层生物标志物指导术后免疫选择。

### 1.2 阴性结果的警示：BR.31与ANVIL研究

辅助免疫治疗的前景并非一片坦途。BR.31研究^[8]^公布了令人失望的结果：在完全切除的IB-IIIA期NSCLC患者中，度伐利尤单抗辅助治疗未能显著改善DFS（主要终点），即使在PD-L1≥25%的预设亚组中亦未观察到获益（HR=0.93, 95%CI: 0.71-1.25, *P*=0.64）。同样，作为美国国家癌症研究所（National Cancer Institute, NCI）ALCHEMIST计划重要组成部分的ANVIL研究^[9]^，其最终结果也未能达到预期：纳武利尤单抗辅助治疗未改善DFS（HR=0.97, *P*=0.39），即使在PD-L1≥50%亚组中亦为阴性结果（中位DFS：89.8 *vs* 78.5个月，HR=0.86，95%CI: 0.59-1.25）。四项标志性III期辅助免疫试验结局存在明显分化：BR.31、ANVIL整体无显著生存获益；IMpower010^[5,6]^、KEYNOTE-091^[7]^仅在特定PD-L1亚组中观察到有限获益（表1^[5-9]^）。

**表1:** 术后辅助治疗临床试验数据汇总

PD-L1 status	IMpower010^[5,6]^	KEYNOTE-091^[7]^	BR.31^[8]^	ANVIL^[9]^
PD-L1≥50%	HR=0.48 (95%CI: 0.32-0.72)	HR=0.82 (95%CI: 0.57-1.18)	HR=0.96 (95%CI: 0.67-1.37)	HR=0.86 (95%CI: 0.59-1.25)
PD-L1 1%-49%	HR=0.87 (95%CI: 0.60-1.26)	HR=0.67 (95%CI: 0.48-0.92)	HR=0.97 (95%CI: 0.74-1.27)	HR=0.98 (95%CI: 0.77-1.25)
PD-L1<1%	HR=0.97 (95%CI: 0.72-1.31)	HR=0.78 (95%CI: 0.58-1.03)	HR=1.04 (95%CI: 0.77-1.41)	HR=0.96 (95%CI: 0.74-1.24)

PD-L1: programmed death ligand 1; HR: hazard ratio; CI: confidence interval.

### 1.3 辅助免疫治疗模式的生物学挑战

上述临床失败并非偶然，其根源在于辅助模式所面临的生物学困境。（1）肿瘤抗原库的缺失：手术切除了原发肿瘤，导致肿瘤抗原缺失。ICIs发挥作用的前提是肿瘤特异性T细胞的识别与活化，而缺乏充足抗原暴露时，T细胞的扩增与克隆选择受到严重限制^[10]^。（2）免疫微环境的破坏：手术不仅移除了肿瘤本体，也破坏了肿瘤引流淋巴结（tumor-draining lymph node, TDLN）及肿瘤微环境（tumor microenvironment, TME），而后者是免疫应答启动和维持的关键场所。辅助阶段缺乏完整的免疫解剖结构，使得ICIs的“免疫激活”无法进行或者显著减弱^[11]^。（3）生物标志物的预测困境：与晚期NSCLC不同，PD-L1表达在辅助模式中的预测价值充满不确定性。IMpower010^[5,6]^中PD-L1≥50%人群显著获益，而KEYNOTE-091^[7]^中同一人群却未获益；BR.31^[8]^和ANVIL^[9]^在所有PD-L1亚组（包括≥50%）均为阴性。这种不一致性揭示了PD-L1无法可靠预测辅助免疫获益，术后组织标本的PD-L1状态可能受手术应激、炎症反应等因素干扰，难以准确反映治疗前的免疫状态。

上述临床证据与生物学机制的共同指向，提示辅助免疫治疗的局限性，也为新辅助模式的推广提供了充分的依据。

## 2 新辅助免疫治疗：围术期的新希望

### 2.1 新辅助模式的生物学优势

与辅助模式相比，新辅助免疫治疗（通常联合化疗）的核心优势在于充分利用了原发肿瘤的存在，其生物学机制可概括为三个层面：（1）肿瘤抗原库的完整性与免疫激活：在原发肿瘤存在时给予免疫联合化疗，化疗诱导的肿瘤细胞死亡释放大量肿瘤抗原，与ICIs协同作用，诱导大规模的肿瘤特异性T细胞浸润和扩增^[12]^。（2）TDLN的参与：新辅助治疗保留了TDLN的解剖完整性，使得抗原呈递细胞（antigen-presenting cell, APC）能够高效地将肿瘤抗原转运至淋巴结，启动系统性抗肿瘤免疫应答^[13]^。（3）组织驻留记忆T细胞（tissue-resident memory T cell, TRM）的诱导：研究^[14,15]^发现，新辅助治疗可诱导高效的TRM形成。这些细胞在术后长期驻留于肺组织及引流淋巴结中，构成持久的局部及全身性免疫监视网络，是预防远期复发的重要机制。

### 2.2 里程碑证据：CheckMate-816研究获得阳性结果

CheckMate-816研究^[16]^是首个在纯新辅助模式下证实总生存（overall survival, OS）获益的III期随机对照试验。该研究将可切除IB-IIIA期NSCLC患者随机分配至纳武利尤单抗联合化疗组（3个周期）或单纯化疗组。结果显示，联合组病理完全缓解（pathological complete response, pCR）率显著提升至24.0%（对照组为2.2%，OR=13.94），中位无事件生存（event free survival, EFS）显著延长（31.6 *vs* 20.8个月，HR=0.63）。更为关键的是，CheckMate-816的5年随访数据^[17]^进一步证实了新辅助免疫治疗的长期生存获益：联合组5年OS率为65.4%，对照组为55.0%，绝对获益达10.4%，死亡风险降低28%（HR=0.72）。该研究首次证实新辅助免疫带来的病理缓解获益可转化为长期总生存改善。值得注意的是，CheckMate-816中达到pCR的患者5年OS率高达95.3%，而未达pCR者仅为55.7%，pCR展现出强大的预后分层价值。后续AEGEAN^[18]^、KEYNOTE-671^[19]^及CheckMate-77T^[20]^等研究进一步验证了新辅助免疫治疗在pCR及EFS方面的优势。

### 2.3 PD-L1在新辅助/围术期模式中的预测价值

与辅助模式的混乱不同，纯新辅助/“夹心饼”模式中，无论PD-L1表达水平如何，免疫联合化疗的效果均优于化疗。CheckMate-816^[16,17]^中，PD-L1≥1%人群EFS HR为0.41，PD-L1<1%人群HR为0.85；CheckMate-77T^[20]^中，PD-L1≥1%人群EFS HR为0.52，PD-L1<1%人群HR为0.73。虽然PD-L1高表达者获益更显著，但即使PD-L1<1%患者，新辅助/围术期免疫治疗仍可带来pCR提升（CheckMate-816^[16,17]^中，PD-L1<1%亚组内纳武利尤单抗联合化疗组与单纯化疗组的pCR率分别为16.7%和2.6%）和EFS获益。这一特征与辅助模式形成鲜明对比，提示PD-L1在新辅助模式中的预测价值较辅助模式更稳定。一项荟萃分析^[21]^也证实：PD-L1表达<1%的患者EFS在接受新辅助治疗后有所改善（HR=0.77, 95%CI: 0.65-0.93），而接受辅助治疗的患者则未观察到这种改善（鳞癌亚组HR=0.93，95%CI: 0.76-1.13；PD-L1<1%亚组HR=0.85，95%CI: 0.71-1.01）。

### 2.4 病理缓解可作为生存替代终点

新辅助治疗模式的一个独特优势在于，手术标本可提供直接的病理疗效评估。pCR（术后标本中无存活肿瘤细胞）和主要病理缓解（major pathological response, MPR）已被证实与长期生存高度相关。CheckMate-816^[17]^中，达到pCR的患者5年OS HR仅为0.11（95%CI: 0.04-0.36），在NADIM研究^[22-24]^中，新辅助纳武利尤单抗联合化疗的pCR率达63.4%，3年OS率为81.9%；其后续NADIM II研究的联合治疗组pCR率为36.8%（化疗组为6.9%），2年无进展生存率为67.2%（化疗组为40.9%，HR=0.47），且达pCR者在随访期间均未出现疾病进展或复发^[22-24]^。国际肺癌研究协会（International Association for the Study of Lung Cancer, IASLC）已就pCR/MPR的标准化评估达成共识，使其成为加速药物开发和临床决策的重要工具^[25]^。

### 2.5 不同治疗模式的对比与思考

随着CheckMate-77T^[20]^、AEGEAN^[18]^及KEYNOTE-671^[19]^等研究的阳性结果陆续公开，“夹心饼”治疗模式也逐渐成为临床指南推荐的标准方案。该模式的理论基础在于：新辅助阶段诱导免疫应答，辅助阶段维持和巩固抗肿瘤免疫，从而实现持续抗癌。然而，这一模式在KEYNOTE-671^[26]^和CheckMate-816^[16]^长期生存数据发布后，也引发了关于治疗强度的深刻反思：（1）单纯新辅助的科学性：若患者在新辅助治疗后已获得pCR，其体内免疫系统已产生极佳的抗肿瘤响应。此时继续追加辅助免疫治疗能否带来额外获益尚缺乏证据支持，反而会增加累积性的免疫相关不良反应（immune-related adverse event, irAE）及经济负担。对于这部分患者，降阶策略可能更为合理。（2）两种新辅助模式的关键数据对比：CheckMate-816（纯新辅助）^[17]^与CheckMate-77T（夹心饼）^[20]^相比，术前3个周期与4个周期的pCR率非常接近（24%和25.3%），提示增加疗程对于pCR的提升非常有限。此外，CheckMate-816与CheckMate-77T的2年EFS率仅相差3.2%（63.8%和67%），与KEYNOTE-671^[26]^（夹心饼）的5年OS率获益也非常相似（65.4%和64.6%），这些长期生存数据提示，辅助阶段的免疫治疗是否必要，值得商榷。不过需要注意的是：CheckMate-816^[16,17]^与KEYNOTE-671^[19]^两项研究入组人群分期、淋巴结负荷基线存在明显差异，需要头对头临床研究最终证实新辅助后的术后辅助免疫的潜在价值（表2^[16,17,19,20,26]^）。（3）新辅助与辅助模式的对比：事实上，目前数据显示辅助和新辅助免疫治疗均有效，但新辅助模式在生物学机制和临床结局上似乎更优于辅助模式。IASLC在2024年的共识中指出^[27]^：对于可切除IIIA/IIIB期患者，新辅助免疫联合化疗优于直接手术（94%支持）；所有II期患者是否都应接受新辅助治疗尚有争议（65%支持）。然而，一项荟萃分析^[21]^却发现在II期NSCLC患者中，新辅助免疫治疗显示出EFS获益（HR=0.69, 95%CI: 0.54-0.88），而辅助免疫治疗未观察到显著的EFS获益（HR=0.81, 95%CI: 0.63-1.05）；在OS层面，新辅助免疫治疗同样具有显著获益（HR=0.65, 95%CI: 0.53-0.81），而辅助免疫治疗则未见显著获益（HR=0.91, 95%CI: 0.76-1.10）。虽然在肺癌领域尚无头对头随机对照试验直接比较两种模式，但是在恶性黑色素瘤领域的SWOG S1801研究^[28]^已证实新辅助联合辅助（围术期）模式在长期生存方面要优于单纯辅助治疗模式。

**表2:** 免疫新辅助III期临床研究结果汇总

Trial name	Neoadjuvant regimen	Neoadjuvant cycles	Postoperative immunotherapy	pCR	2-yr EFS	5-yr EFS	5-yr OS
CheckMate-816^[16,17]^	Nivolumab plus chemotherapy	3	No	24%	63.8%	49%	65.4%
CheckMate-77T^[20]^	Nivolumab plus chemotherapy	4	Yes	25.3%	67%	Follow-up ongoing	Follow-up ongoing
KEYNOTE-671^[19,26]^	Pembrolizumab plus chemotherapy	4	Yes	18.1%	62.4%	49.9%	64.6%

pCR: pathological complete response; EFS: event free survival; OS: overall survival.

## 3 精准未来：基于pCR与MRD的个体化决策模型

### 3.1 pCR：局部疗效评价指标，无法反映全身疾病状态

pCR是新辅助治疗最重要的短期疗效评价指标，具有强大的预后分层价值。CheckMate-816^[17]^中，pCR患者5年OS率高达95.3%，显著优于non-pCR患者的55.7%。然而，pCR作为单一指标存在明显局限：其一，pCR/Non-pCR的二元划分忽略了肿瘤残留程度的信息；其二，pCR仅反映手术标本的局部病理状态，而非全身免疫/肿瘤状态；其三，即使达pCR患者仍有复发风险。这一局限性在CheckMate-816的病理探索性分析中得到充分揭示——该研究采用pan-tumor评分系统对残留活肿瘤细胞百分比（residual viable tumor cell percentage, % RVT）进行连续定量评估，发现RVT与EFS呈显著线性相关：每增加1% RVT，EFS风险比增加0.017（HR=1.017）。RVT的预后分层远较pCR/MPR的二元更精细：免疫联合化疗组中，2年EFS率随RVT升高呈阶梯式下降：0%-5% RVT者为90%，5%-30%者为60%，30%-80%者为57%，>80%者为39%；RVT预测2年EFS的曲线下面积（area under the curve, AUC）达0.74，优于影像学缓解和循环肿瘤DNA（circulating tumor DNA, ctDNA）清除^[29]^。

### 3.2 分子残留病灶（molecular residual disease, MRD）：全身疾病监测指标，pCR的重要补充

MRD检测通过ctDNA分析，能够在影像学复发前数月甚至数年识别亚临床残留病灶，是指导术后个体化治疗的新兴工具^[30]^。与pCR相比，MRD具有三大核心优势：评估范围为全身性而非局部性；时间特性为动态可反复监测而非静态一次评估；检测方法为液体活检而非病理学评估。NADIM II研究的探索性分析^[31]^显示，术后MRD阳性与极差的预后显著关联：MRD阳性患者的EFS和OS HR分别高达10.2（95%CI: 3.7-28.3）和10.0（95%CI: 2.0-49.9）。尤为重要的是，MRD还可增强pCR的预后分层价值：即使达pCR者，若术后MRD阳性，其预后也可能劣于MRD持续阴性的non-pCR患者。而在non-pCR人群中，MRD也是显著预后标志，至少两次MRD检测均为阴性的患者，其生存结局优于MRD阳性或持续阳性的患者。这提示pCR与MRD联合评估可构建更为精细的风险分层体系^[31]^。MERMAID-1（NCT04385368）为国际多中心III期随机双盲试验，针对根治术后MRD阳性的II-III期NSCLC人群，对比度伐利尤单抗联合标准辅助化疗与安慰剂，旨在验证MRD阳性高危患者术后免疫升阶强化治疗的生存获益；ADOPT-lung（NCT06284317）则入组新辅助免疫联合化疗后non-pCR的IIB-IIIB（N2）期可切除患者，随机对比术后度伐利尤单抗辅助巩固与单纯观察，明确残留病灶人群追加辅助免疫的临床价值。基于pCR与MRD各自的优势与局限，二者联合运用有望构建更精准的临床决策框架。

### 3.3 个体化决策框架

基于现有证据，我们认为：纯新辅助治疗模式是优选，3个周期治疗后对于pCR且MRD阴性者采取观察策略（降阶），因两者均提示亚临床病灶负荷极低、复发风险可控，可避免不必要的辅助治疗。non-pCR和/或MRD阳性者则转向辅助强化治疗（升阶）。当前，ADOPT-lung研究（NCT06284317）正探索non-pCR人群中辅助度伐利尤单抗对比观察的价值；此外，针对non-pCR患者，术后放疗（NCT06878274）^[32]^以及靶向滋养层细胞表面抗原2（trophoblast cell surface antigen 2, TROP2）的抗体偶联药物（antibody-drug conjugate, ADC）（NCT06312137）^[33]^的升阶梯治疗的临床研究也在进行。

上述决策模型的核心逻辑在于，围术期免疫治疗的目标并非最大化治疗强度，而是最大化治愈概率。传统围术期模式通常将所有患者视为同质人群，采用统一的辅助免疫策略。然而，新辅助治疗后的患者具有高度异质性：部分患者已获得pCR并实现MRD清除，提示体内肿瘤负荷极低，继续辅助治疗可能仅带来有限的获益；而另一部分患者存在病理残留或MRD持续阳性，则提示潜在耐药克隆或微转移灶仍然存在，需要进一步强化治疗。因此，未来围术期免疫治疗的发展方向，可能并非“所有患者接受更长时间治疗”，而是基于病理缓解与分子残留状态实施风险适配的精准干预（图1）。

**图1:**
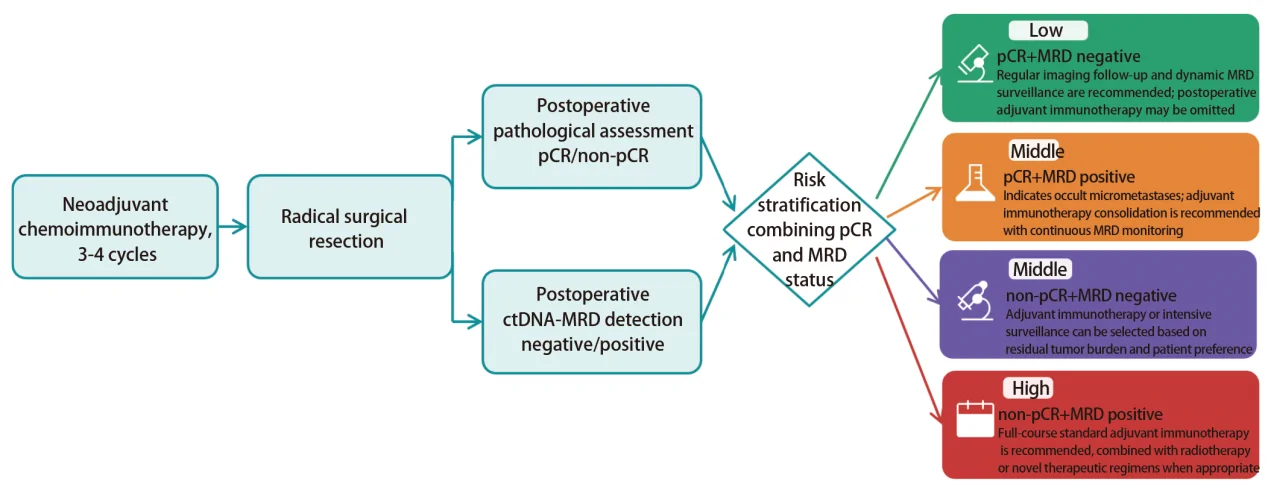
基于pCR+MRD双维度的临床决策流程图

## 4 卫生经济学考量

成本效益问题也是临床诊疗中的重要议题^[34]^。在卫生经济学评价中，国际通行的方法主要包括成本效果分析（cost-effectiveness analysis, CEA）与成本效用分析（cost-utility analysis, CUA）。其中，CUA通常以质量调整生命年（quality-adjusted life years, QALY）作为统一效益指标，并计算增量成本效果比（incremental cost-effectiveness ratio, ICER），即：ICER=（新方案成本-对照方案成本）/（新方案QALY-对照QALY）。随后将ICER与各国支付意愿阈值（willingness-to-pay threshold, WTP）比较，以判断是否具有“经济可接受性”。例如，美国常用阈值为50,000-150,000 美元/QALY，而英国国家卫生与临床优化研究所（National Institute for Health and Care Excellence, NICE）体系通常采用20,000-30,000英镑/QALY。White等^[35]^的成本效果分析显示，围术期“夹心饼”方案（CheckMate-77T）对比纯新辅助方案（CheckMate-816），追加术后纳武利尤单抗带来的生存获益增幅有限，而治疗成本显著增加，从而导致ICER升高至153,557美元/QALY，成本效益相对降低。在美国这一数值可能处于边际可接受区间，但在中低收入国家通常不具备成本效益优势。按中国市场计算，CheckMate-816方案（术前3个周期纳武利尤单抗约8.3万元）较CheckMate-77T（术前+术后1年纳武利尤单抗约25万元）和KEYNOTE-671（术前+术后1年帕博利珠单抗约48万元）的药物成本分别降低约67%和83%。因此，在资源有限地区，采用“纯新辅助”的CheckMate-816模式可能更好地提高每单位医疗支出的健康产出。此外，印度两项探索性研究^[36,37]^提示低剂量免疫治疗策略可在维持疗效的同时降低治疗成本，为资源受限地区提供了治疗参考。特瑞普利单抗^[38]^和替雷利珠单抗^[39]^的围术期适应证已纳入我国医保，但是均采用“夹心饼”的围术期给药模式。后续可鼓励国产程序性死亡受体1（programmed death 1, PD-1）/PD-L1抑制剂开展“纯新辅助”治疗模式的临床研究，在保证长期生存获益的前提下探索短疗程方案，提升治疗方案的卫生经济学价值，减轻患者与医保基金的长期医疗负担。

## 5 总结与展望

综上，可切除NSCLC的围术期管理呈现出“新辅助优先”的趋势。辅助免疫治疗因BR.31^[8]^、ANVIL^[9]^等研究的阴性结果而陷入困境，其生物学局限性，如肿瘤抗原暴露不足、既有免疫微环境破坏及PD-L1预测价值不稳定，提示其难以成为最佳围术期策略。相比之下，新辅助免疫治疗凭借完整的抗原暴露、系统性免疫激活及TRM诱导等机制优势，在CheckMate-816等^[16,17]^诸多研究中已显示出显著pCR率提升及长期生存获益。在此背景下，“纯新辅助”策略在达到相似疗效的前提下，较“夹心饼”模式可能更具治疗效率与潜在性价比，尤其适用于实现pCR的患者。

需要强调的是，“新辅助优先”策略的适用人群应有明确边界。其一，从分期来看，纯新辅助模式的关键证据（CheckMate-816）主要来自可切除IB-IIIA期人群，而“夹心饼”模式（CheckMate-77T、KEYNOTE-671）的证据主要来自II-IIIB（N2）期人群，IB期低危患者接受新辅助免疫的绝对获益尚不明确，应结合肿瘤大小、淋巴结状态等个体化权衡^[16,19,20]^。其二，现有新辅助免疫III期研究均纳入表皮生长因子受体（epidermal growth factor receptor, *EGFR*）/间变性淋巴瘤激酶（anaplastic lymphoma kinase, *ALK*）野生型患者，驱动基因阳性人群被排除或证据不足，此类患者仍应以相应的靶向治疗为主，不宜直接套用“新辅助优先”策略^[16,19,20]^。其三，患者需具备可耐受免疫联合含铂双药化疗的体能状态与脏器功能储备，即美国东部肿瘤协作组（Eastern Cooperative Oncology Group, ECOG）体能状态评分0-1分；合并活动性自身免疫性疾病、间质性肺病，或需长期使用糖皮质激素/免疫抑制剂者，应慎用或避免ICIs治疗。因此，临床实践中应在多学科评估的基础上，综合分期、驱动基因状态、体能状态及免疫治疗禁忌证确定“新辅助优先”策略的适用边界，以期在保证安全性的前提下最大化围术期免疫治疗的获益。

然而，上述“新辅助优先”策略及疗效优势的解读仍需谨慎。一方面，现有对辅助与新辅助模式疗效差异的判断主要基于不同III期研究之间的间接比较（如CheckMate-816^[17]^、CheckMate-77T^[20]^、KEYNOTE-671^[26]^），各研究在入组分期、化疗方案、治疗周期及PD-L1检测平台等方面均存在差异，使跨研究比较不可避免受到人群异质性与选择偏倚影响。另一方面，MRD作为疗效分层与治疗决策工具虽具有重要前景，但其检测技术尚未标准化，包括检测panel构成、采样时间点及阳性阈值等均缺乏统一标准。目前相关证据多来自NADIM II^[23]^、TRACERx^[30]^等研究的探索性分析或II期试验，仍缺乏前瞻性随机验证，其临床决策价值需等待MERMAID系列等III期研究进一步确立。此外，当前卫生经济学评价（如ICER分析）多基于模型推演，其参数设定主要参考欧美医疗体系，在中国真实临床路径与医保支付环境下的适用性仍存在一定局限。
